# A Picture Worth a Thousand Molecules—Integrative Technologies for Mapping Subcellular Molecular Organization and Plasticity in Developing Circuits

**DOI:** 10.3389/fnsyn.2020.615059

**Published:** 2021-01-05

**Authors:** Jacqueline A. Minehart, Colenso M. Speer

**Affiliations:** Department of Biology, University of Maryland, College Park, MD, United States

**Keywords:** connectomics, local protein translation, neural development, proteomics, super-resolution microscopy, transcriptomics, translatomics, synaptic plasticity

## Abstract

A key challenge in developmental neuroscience is identifying the local regulatory mechanisms that control neurite and synaptic refinement over large brain volumes. Innovative molecular techniques and high-resolution imaging tools are beginning to reshape our view of how local protein translation in subcellular compartments drives axonal, dendritic, and synaptic development and plasticity. Here we review recent progress in three areas of neurite and synaptic study *in situ*—compartment-specific transcriptomics/translatomics, targeted proteomics, and super-resolution imaging analysis of synaptic organization and development. We discuss synergies between sequencing and imaging techniques for the discovery and validation of local molecular signaling mechanisms regulating synaptic development, plasticity, and maintenance in circuits.

## Introduction

In neuroscience, physical maps of synaptic connections between neurons (“connectomes”) help to refine hypotheses about neural computation and provide a shared anatomical resource to guide investigations of circuit function and pathologies associated with human brain disease. Physical maps of developing circuits can also help to identify progressive structural changes across stages of neuronal and synaptic maturation. Connectomic maps are produced using volumetric electron microscopy (EM) techniques that provide dense reconstructions of all cellular processes and synaptic connections in brain tissue. Continued progress in automated sample collection, high-throughput imaging, and computational analysis (Kornfeld and Denk, 2018; Kubota et al., 2018; Motta et al., 2019b) will enable routine investigation of connectomes by EM, thereby creating anatomical maps of common model systems for all neuroscientists to use in research (White et al., 1986; Kim et al., 2012; Helmstaedter et al., 2013; Lee et al., 2016; Morgan et al., 2016; Eichler et al., 2017; Hildebrand et al., 2017; Zheng et al., 2018; Cook et al., 2019; Motta et al., 2019a; Abbott et al., 2020; Scheffer et al., 2020; Witvliet et al., 2020).

While connectomic analysis has advanced significantly in recent decades, most synapse-level diagrams do not contain molecular information important for understanding circuit development or synaptic function at maturity (Bargmann and Marder, 2013). Synapses formed by different cell types are molecularly diverse and this diversity contributes to microcircuit differences in neurotransmission as well as synaptic connectivity rules during brain development (O’Rourke et al., 2012; Paul et al., 2017; Nusser, 2018; Grant and Fransén, 2020). Thus, it is important to complement and support EM-based connectomic maps with “molecular connectomic” information to gain a better understanding of how circuits are formed and how particular synaptic connections contribute to computation and behavior (Schreiner et al., 2017). Relevant molecular information includes the abundance and species of proteins (proteome) as well as the local population of RNAs that are trafficked (transcriptome) and translated (translatome) in subcellular compartments. Changes in the local transcriptome, translatome, and proteome each contribute to circuit development at multiple spatial scales from individual synapses to dendritic/axonal architecture, ultimately leading to mature network development (Cioni et al., 2018; Biever et al., 2019; Holt et al., 2019).

**Figure 1:**
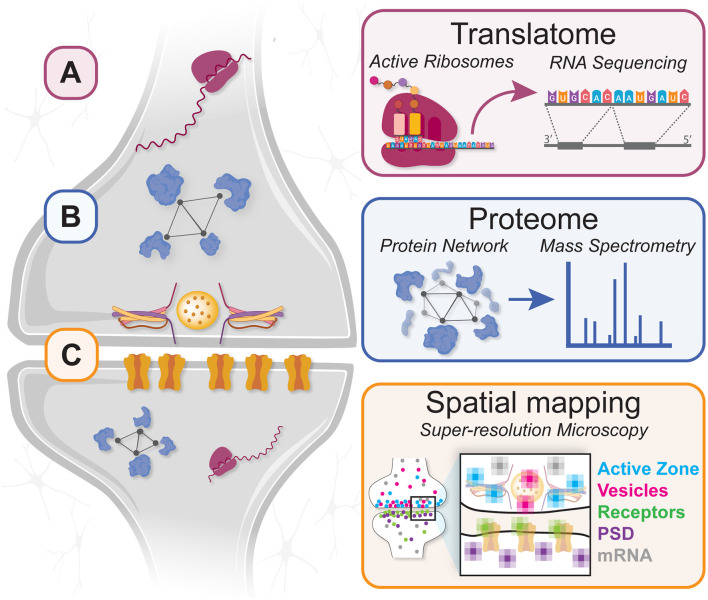
A “molecular connectomic” combination of techniques for investigating local protein translation and synaptic proteome plasticity during circuit development. **(A)** Affinity purification techniques enable RNA-seq analysis of the local translatome to identify translating mRNAs in developing neurites/synapses. **(B)** Proximity labeling and nascent protein tagging methods enable mass spectrometry analysis of synaptic proteomes in developing circuits. **(C)** Spatial mapping by super-resolution microscopy provides nanoscale information about protein and mRNA locations in developing axons, dendrites, and synapses.

In this review article, we discuss a bottom-up “developmental molecular connectomics” approach for investigating the regulation of local molecular remodeling during circuit refinement (Figure 1). We highlight recent progress in three research areas that together are expanding our understanding of neural development, synaptic refinement, and activity-dependent circuit plasticity *in situ*: (1) molecular-genetic approaches for quantifying mRNA diversity, abundance, and regulation of the local transcriptome/translatome in targeted subcellular compartments; (2) proteomic techniques for labeling, isolating, and quantifying nascent proteins and protein-protein interaction networks in specific synaptic connections; and (3) super-resolution fluorescence microscopy for multi-scale imaging and quantitative analysis of subsynaptic molecular organization within developing microcircuits. When applied together, advanced techniques in these areas have important synergies that facilitate discovery, validation, and throughput in new experiments designed to study the local molecular regulation of neural circuit development.

## Investigating Local Protein Translation in Targeted Circuits

Neural circuit development requires the precise formation and selective plasticity of synaptic connections distributed across each neuron’s polarized geometry (axons/dendrites) at significant distances from the soma. Because each synapse is an intricate molecular machine assembled from thousands of proteins, the development of circuit connections creates a significant metabolic demand for individual neurons (Cohen et al., 2013). Proteins destined for synaptic localization are either trafficked from the soma by motor-driven transport (Guedes-Dias and Holzbaur, 2019; Radler et al., 2020) or translated directly in subcellular compartments from pools of trafficked mRNAs. Local protein translation near synapses may offer several advantages including increased metabolic efficiency, precise spatiotemporal delivery of nascent proteins to synaptic connections, and site-dependent regulation of posttranslational protein modification unique to synaptic compartments (Spaulding and Burgess, 2017; Cioni et al., 2018; Van Driesche and Martin, 2018; Biever et al., 2019). Consistent with these benefits, local protein translation is involved in many if not all aspects of neural circuit development including neurite guidance, axonal and dendritic branching, synaptogenesis, synaptic plasticity, and neural health (Holt et al., 2019).

Because local protein production depends on local mRNAs, an effective bottom-up approach to the problem begins with the analysis of cell-type-specific mRNA pools isolated from subcellular compartments such as axons, dendrites, and synapses. To link the local transcriptome to downstream changes in the proteome, researchers have also developed a family of techniques for exploring the translatome, a subset of mRNAs that are actively engaged with ribosomes during protein translation. Combined with cell-type-specific transgenic labeling approaches, these tools enable the sequencing of circuit-specific transcripts purified from subcellular compartments in developing circuits.

### Mapping Local Transcriptomes/Translatomes in Circuits

**Figure 2:**
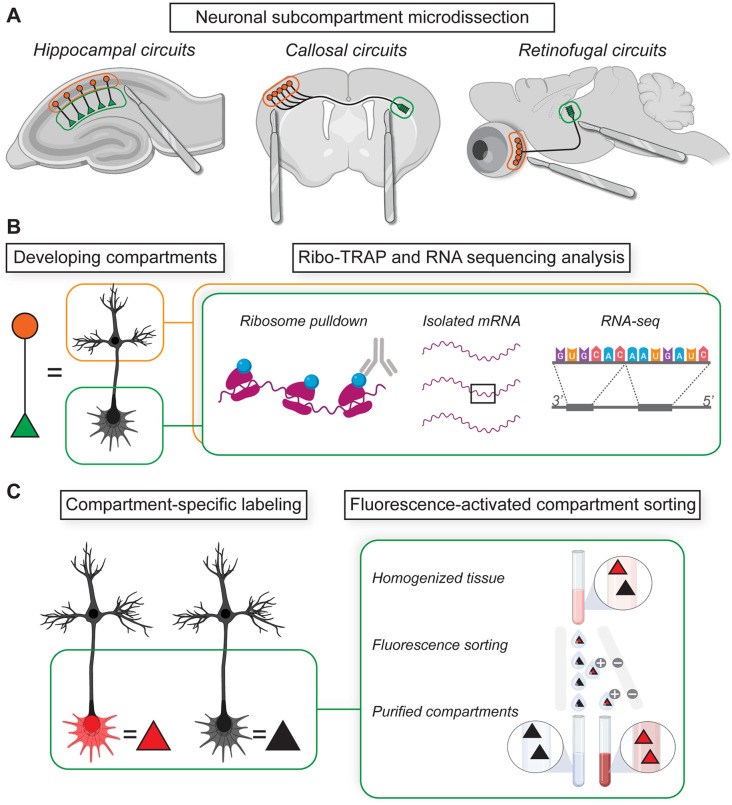
Isolating local mRNAs and proteins from subcellular compartments. **(A)** Physical microdissection enables the separation of cell bodies from distal neurites in classic model circuits for investigating neural development. **(B)** Locally translated mRNAs in subcompartment fractions can be isolated and sequenced using translating ribosome affinity purification (TRAP) methods. **(C)** Transgenic labeling methods enable fluorescence-activated compartment sorting for RNA and protein sequencing analyses.

A straightforward approach to quantify mRNAs in subcellular compartments is to physically separate the different parts of a cell (e.g., dendrites, axons, and soma) and perform RNA sequencing analysis of compartment-specific isolates for comparison (Figure 2A). Such experiments have been pioneered using the hippocampus as a model, where the spatial segregation of pyramidal cell bodies and their neurites enables targeted imaging, microdissection, and biochemical analysis of synaptic neuropil. Remarkably, hippocampal neuropil contains thousands of transcript species and many synapse-related transcripts are differentially enriched in neurites relative to their parent somata (Cajigas et al., 2012; Farris et al., 2019; Wang et al., 2019; Glock et al., 2020; Ohashi and Shiina, 2020). More than 4,800 unique mRNA species are translated in this area and ~16% of these transcripts, many of which encode proteins implicated in neurodevelopmental disorders, are selectively enriched for translation in dendrites (Glock et al., 2020). In support of circuit-specific dendritic trafficking of unique transcripts, targeted laser capture microdissection combined with RNA-seq analysis has shown that unique hippocampal dendritic fields are distinguished by their distinct mRNA species and abundance patterns (Farris et al., 2019). This spatial organization raises questions about how mRNAs are selectively trafficked into subcellular compartments and how changes in mRNA species/abundance relate to synaptic development (Cioni et al., 2018).

To address questions about directed trafficking and mRNA enrichment in specific spatiotemporal contexts, an immunopurification approach called Translating Ribosome Affinity Purification (TRAP) was developed for isolating genetically-tagged ribosomes and their associated mRNAs in targeted circuits (Doyle et al., 2008; Heiman et al., 2008; Sanz et al., 2009). TRAP methods work by isolating affinity-tagged ribosomes (e.g., tagged with HA or GFP) in the presence of compounds that stall ribosome translocation, thereby enabling the purification of mRNAs associated with ribosomes. TRAP technology has inspired innovative applications that form a flexible “toolkit” for cell-type and compartment-specific analysis of local translatomes in developing neurons (Dougherty, 2017). One such tool, the “axon-TRAP” approach (discussed further in following sections), uses transgenic labeling of axonal projections to enable cell-type and axon-specific analyses of developing translatomes during pathfinding, ingrowth, branching, and synaptogenesis (Shigeoka et al., 2016; Figure 2B). Also in the toolkit are dozens of bacTRAP mouse lines for labeling ribosomes in select cell populations and brain areas (Doyle et al., 2008) as well as a floxed RiboTag transgenic for cell-type-specific translatome purification in cre-recombinase driver lines (Sanz et al., 2009). TRAP methods have also revealed compartmentalized local translation contributing to gliovascular signaling at astrocytic endfeet (Boulay et al., 2017) as well as at tripartite synapses in the brain (Sakers et al., 2017; Mazaré et al., 2020).

In addition to microdissection and affinity purification methods, Fluorescence-Activated Synaptosome Sorting (FASS) uses the transgenic expression of synapse-specific fluorescent fusion proteins to purify specific synapse types from total synaptosomal fractions based on fluorescence-activated cell sorting (FACS; Biesemann et al., 2014; Luquet et al., 2017; Figure 2C). FASS has helped characterize the local transcriptome of VGluT1(+) excitatory presynaptic terminals isolated from mature cortical, hippocampal, and cerebellar circuits *in situ* (Hafner et al., 2019). These excitatory terminals are enriched for transcripts encoding synaptic proteins, ribosomal proteins, mitochondrial proteins, and initiation/elongation factors known to play important roles in the regulation of mRNA translation (Hafner et al., 2019). For investigating neurite development *in situ*, a related fluorescence-sorting approach was developed to purify the local transcriptome of developing Layer 2–3 cortical axonal growth cones during the formation of the anterior commissure (Poulopoulos et al., 2019). After separating fluorescent-labeled growth cones from the bulk tissue, mRNA and protein networks were analyzed in the same samples to achieve an elegant comparison of distal transcriptomes and proteomes during axon pathfinding (Poulopoulos et al., 2019). Such fluorescence-sorting techniques have revealed a high level of specificity in transcript trafficking to subcellular compartments consistent with regulated mRNA trafficking being the *sine qua non* of local protein translation.

### Local Transcriptomes/Translatomes Are Precise

**Figure 3:**
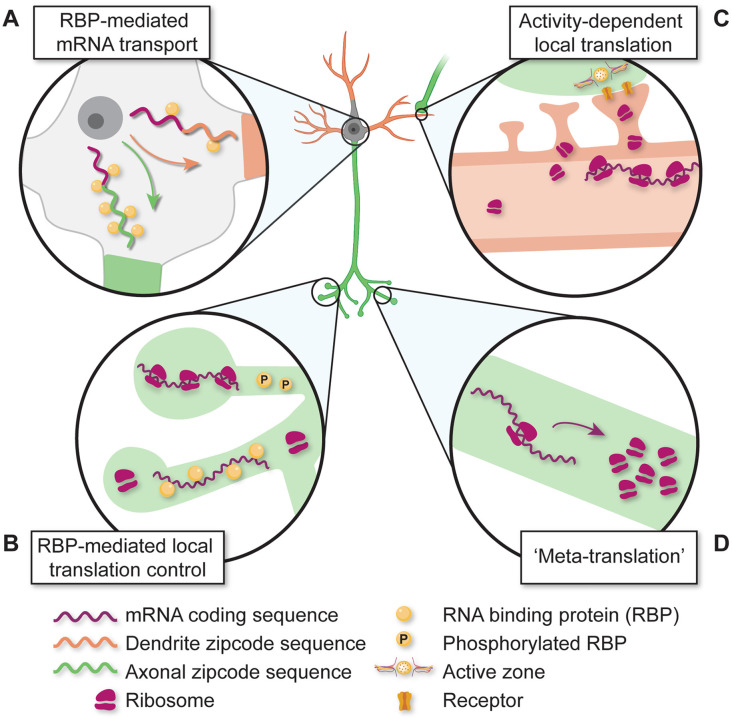
Diverse mechanisms for regulating local translation in developing subcompartments. **(A)** mRNAs are differentially transported based on RNA-binding protein (RBP) interactions with unique non-coding sequences. **(B)** Dynamic interactions between RBPs and mRNAs regulate local translation. **(C)** Synaptic inputs and neuronal activity regulate local protein translation and the accumulation of translation machinery. **(D)** The local translation of protein synthesis machinery regulates local protein translation activity in neurites and synapses.

Microdissection and TRAP approaches for isolating RNA have revealed important roles for transcript UTR sequence motifs and transcript trafficking proteins in local translation. mRNAs are transported in phase-separated ribonucleoprotein (RNP) granules over long distances to dendrites and axons to establish local transcriptome pools (Nalavadi et al., 2012; Jung et al., 2014; Das et al., 2019; Liao et al., 2019; Pushpalatha and Besse, 2019; Fukuda et al., 2020; Rhine et al., 2020; Wu et al., 2020; Figure 3). The spatial control of transcript trafficking is based on mRNA sequences found in 3′ and 5′ untranslated regions (UTRs; Figure 3A) that act as binding sites for *trans-*acting RBPs (Sahoo et al., 2018; Bae and Miura, 2020). In turn, RBP-RNA binding regulates RNA trafficking/localization, stability, and translation (Hentze et al., 2018; Figure 3B). As a consequence of these critical roles, dysregulations in RNA-RBP interactions are implicated in neurodegenerative diseases (Pushpalatha and Besse, 2019; Thelen and Kye, 2020).

To investigate the role of 3′-UTR regulatory sequences in mRNA trafficking *in situ*, mRNA from hippocampal somatic and neuropil layers was isolated and 3′ end sequenced to identify compartment-specific differences in transcript isoforms (Tushev et al., 2018). Neuropil-enriched transcripts have significantly longer 3′-UTRs with multiple regulatory domains recognized by RNA binding proteins and miRNAs (Ouwenga et al., 2017; Tushev et al., 2018; Farris et al., 2019). Longer 3′-UTRs increase the opportunity for RBP interactions that stabilize mRNAs in granules, reduce transcript translation efficiency, and enable long-range mRNA trafficking. As a group, transcripts with long 3′-UTRs are translated with lower efficiency than transcripts with shorter 3′-UTRs (Blair et al., 2017; Glock et al., 2020; Rodrigues et al., 2020). A similar role for 3′-UTR translational regulation has also been reported in glia, where TRAP analysis of translatomes from peripheral astrocytic processes revealed enrichment of mRNAs with long 3′-UTRs and common *cis*-elements for RBP-mediated mRNA stabilization and transport (Sakers et al., 2017). The importance of mRNA half-life and the 3′-UTR for new protein production in distal compartments is further supported by computational models that predict significant decreases in protein distribution in distal neurites when mRNA half-life is decreased or 3′-UTR regions are deleted (Fonkeu et al., 2019). These data highlight the importance of alternative 3′-UTR isoforms as a mechanism for dynamically adjusting mRNA stability and translation.

Similar to the regulatory structure of the 3′-UTR, 5′-UTR *cis*-elements also have important roles in regulating local protein translation essential for brain development. During laminar development of the neonatal cortex, 5′-UTR-dependent mRNA trafficking and local translation regulate radial glial cell branching and neuronal migration patterns to establish proper cortical lamination (Pilaz et al., 2020). At later developmental stages, 5′-UTR cis-sequences have been implicated in local translation underlying axon growth/pathfinding. Callosal axon growth cones purified from the tissue by fluorescence sorting are enriched with transcripts containing a 5′ terminal oligopyrimidine (TOP) motif (Poulopoulos et al., 2019), a regulatory sequence essential for mTOR-dependent translation (Thoreen et al., 2012). mTOR complex proteins are also enriched in growth cones and disruption/knock-out of mTOR activation causes defects in callosal axon pathfinding (Poulopoulos et al., 2019). Interestingly, mTOR protein is locally translated in axons, and disrupting axonal mTOR mRNA localization by 3′-UTR deletion leads to decreased protein translation and neuronal loss in a nerve injury model (Terenzio et al., 2018). These data suggest that “meta-translation”—the local translation of key regulators of local protein translation (e.g., mTOR, others)—may serve to amplify compartment-specific protein translation (Figure 3D). Consistent with this, axonal mRNAs containing unique 5′-UTR regulatory sequences also encode ribosomal proteins and regulators of translation initiation, implicating the local control of ribosome abundance, composition, and activity in the normal emergence of callosal connections (Poulopoulos et al., 2019) as well as retinal axon projections to central brain targets in the developing visual system (Shigeoka et al., 2016, 2019).

Together TRAP, FASS, and other compartment-specific isolation experiments highlight the precise nature of local translation and pave the way for the continued study of its regulation. Checkpoints under investigation include translation initiation controlled by regulatory sequences in the 5′-UTR (Hinnebusch et al., 2016; Leppek et al., 2018), changes in the assembly and composition of ribosomes (Klinge and Woolford, 2019), control of ribosome processivity during elongation (Schuller and Green, 2018), and posttranscriptional modifications of mRNAs to control their stability, structural folding, and engagement with ribosomes (Tian and Manley, 2016; Frye et al., 2018; Ganser et al., 2019; Zaccara et al., 2019). These diverse controls may enable considerable flexibility in the regulation of local protein production to guide progressive stages of neural circuit development.

### Local Transcriptomes/Translatomes Are Remodeled During Circuit Development

To enable rapid spatiotemporal responses to environmental cues, neurite, and synaptic development is regulated in part by local signaling events including local protein translation. Axon-TRAP experiments in the developing visual system have shown that the local translatome in retinal ganglion cell (RGC) axons is developmentally-regulated and shifts during different stages of circuit refinement (Shigeoka et al., 2016; Figures 2A,B). In the mouse, the immature RGC axonal translatome is enriched with mRNAs encoding proteins involved in axon outgrowth, guidance, and branching (Shigeoka et al., 2016). As synaptogenesis proceeds during circuit maturation, the local RGC axonal translatome shifts away from the production of proteins regulating neurite-growth toward transcripts encoding proteins essential for synaptic transmission (Shigeoka et al., 2016). These results raise an important question— what mechanisms regulate the developmental remodeling of compartment-specific translatomes? One possibility is the stage-specific regulation of axonal vs. dendritic mRNA trafficking resulting from the expression of particular RBPs. For example, the association of the RBPs Pumilio (Pum) 1 and 2 with mRNAs containing 3′-UTR Pumilio binding elements (PBEs) prevents these transcripts from entering axons during development (Shigeoka et al., 2016; Martínez et al., 2019). Genetic loss of Pum2 causes many mRNAs with PBEs to mislocalize into developing axons and Pum2 knock-down mice have impaired axon outgrowth and branching during postnatal callosal pathway development (Martínez et al., 2019).

Another mechanism for compartment-specific translatome remodeling is ribosome protein synthesis (Figure 3D). Biochemical purification of mRNAs from the tissue has consistently demonstrated compartment-specific expression of transcripts encoding ribosomal proteins (Cajigas et al., 2012; Shigeoka et al., 2016; Farris et al., 2019; Hafner et al., 2019; Ostroff et al., 2019; Poulopoulos et al., 2019; Mazaré et al., 2020) whose local translation is upregulated in response to extracellular cues (Shigeoka et al., 2019). Metabolic labeling of newly synthesized proteins reveals that locally produced ribosomal proteins are incorporated into existing axonal ribosomes, consistent with a role in the maintenance or selective posttranslational modification of ribosomal machinery in developing neurites (Shigeoka et al., 2019). As circuits mature, early-stage developmental roles for local protein synthesis are fulfilled and synaptogenic signals reduce local ribosome pools to decrease local protein synthesis (Costa et al., 2019). Synaptic maturation may act as a brake on the local translation of branching-related transcripts to constrain neurite elaboration following synapse formation and strengthening (Vaughn, 1989; Cline and Haas, 2008).

Concurrent with synaptic and neurite remodeling, mitochondrial dynamics play an important role in local translation. Recent biochemical and imaging experiments indicate that ATP production fuels local protein translation in subcellular compartments (Cioni et al., 2019; Rangaraju et al., 2019). In a complementary way, axon-TRAP and compartment-specific biochemical isolation experiments *in situ* show local protein translation is itself important for mitochondrial function. mRNAs associated with mitochondrial function and energy homeostasis are locally translated in neurites (Yoon et al., 2012; Kratz et al., 2014; Shigeoka et al., 2016; Gale et al., 2018; Poulopoulos et al., 2019) where they colocalize with RBPs and ribosomes in local translation hubs within axons (Cioni et al., 2019). These local translation platforms are found adjacent to axonal mitochondria where they help produce new proteins essential for mitochondrial health and function (Cioni et al., 2019).

Similar results have been shown in purified synapses (synaptosomes) where mRNAs encoding essential respiratory chain complex proteins are locally translated and assembled into mitochondria in response to stimulation (Kuzniewska et al., 2020). Axon-TRAP experiments have further identified that lamin B2 (LB2) mRNA is translated in axons and inhibition of local LB2 synthesis impairs mitochondrial fission and axon health during development (Yoon et al., 2012). Mitochondrial fission at nascent axon branch points also provides the ATP necessary for local translation of proteins that regulate actin remodeling and axon branching (Spillane et al., 2012, 2013; Lewis et al., 2018; Armijo-Weingart et al., 2019).

In addition to their roles in developing axons, mitochondria are essential for postsynaptic plasticity mechanisms in dendrites. Local protein synthesis in dendrites is powered by mitochondria (Rangaraju et al., 2019) and the induction of long-term potentiation (LTP) in mature synapses requires mitochondrial fission to provide sufficient ATP for new protein synthesis underlying synaptic plasticity (Divakaruni et al., 2018). Because synaptic activity and function are tightly coupled to synaptic ATP production (Rangaraju et al., 2014) and local protein synthesis (Scarnati et al., 2018), activity-dependent synaptic refinement during development may involve a coordinated regulation of mitochondrial position/function with local protein translation to enhance neurite branching and synaptic maturation.

### Local Protein Translation Underlies Activity-Dependent Plasticity

The most well-studied example of activity-dependent local protein translation is that which occurs during the induction of LTP/depression (LTP/LTD). The induction of hippocampal LTP/LTD *in situ* by molecular signals such as neurotrophins, brain-derived neurotrophic factor, metabotropic glutamate receptor agonists, or by electrical stimulation is dependent on local protein synthesis (Kang and Schuman, 1996; Huber et al., 2000; Younts et al., 2016). Such signaling factors drive differential remodeling of local protein translation in distinct subcellular compartments such as dendritic shafts vs. spines suggesting that local protein translation may be fine-tuned at the synaptic scale by unique activity patterns and signaling pathways (Hafner et al., 2019; Figure 3C).

Direct evidence for local (synaptic) protein production in response to synaptic stimulation comes from glutamate photouncaging experiments which drive spine growth and local protein synthesis in single activated spines (Yoon et al., 2016; Rangaraju et al., 2019). In addition to its importance in Hebbian plasticity, local protein translation contributes to homeostatic plasticity as elevated neuronal activity levels cause compartment-specific changes in transcript isoform abundance and an overall shortening of transcript 3′-UTRs (Tushev et al., 2018). Changes in the 3′-UTR length could reflect mRNA translocation between compartments, splicing (shortening) of mRNAs, and/or modulation of RNA stability/degradation to help control local translation rates in subcellular compartments (Tushev et al., 2018; Farris et al., 2019). Activity-dependent global shortening of 3′-UTRs in hippocampal neurite transcripts has also been observed following LTP induction (Fontes et al., 2017), consistent with an increase in local translation during synaptic plasticity.

In addition to the experimental induction of LTP/LTD, natural learning can alter the selective regulation of transcript levels. Mice experiencing novel environmental enrichment show increases in immediate, early gene transcripts in synaptic neuropil with transcript levels further regulated by translation-dependent degradation (Farris et al., 2014). The relevance of local protein translation for learning and memory *in vivo* is supported by experiments showing that late phase hippocampal LTP and spatial/contextual memory performance are each impaired by manipulations of 3′-UTR sequences that prevent dendritic localization and translation of calcium/calmodulin-dependent protein kinase II (CamKII; Miller et al., 2002). More recent TRAP experiments reveal that fear conditioning drives increased ribosome binding to mRNA in CA1 neurites of the hippocampus (Ainsley et al., 2014) and also modulates local translation in peripheral glial processes isolated from tripartite synapses (Mazaré et al., 2020). Further, axon-TRAP has been used to investigate changes in the local translatome of auditory cortical axons projecting to the lateral amygdala. Following auditory fear conditioning, memory consolidation drives increased axonal expression of transcripts encoding mitochondrial proteins and regulators of protein translation such as ribosomal proteins and elongation/initiation factors (Ostroff et al., 2019). These results provide evidence of experience-dependent local “meta-translation” (Figure 3D) during learning and suggest that changes in the axonal translatome may contribute to bidirectional synaptic plasticity in the mature amygdala (Ostroff et al., 2010, 2012).

To examine the ultrastructural basis of protein synthesis-dependent synaptic plasticity, volumetric serial-section electron microscopy has shown that hippocampal LTP induction increases polysome association with enlarged dendritic spines, demonstrating that protein translation machinery is subject to activity-dependent remodeling during synaptic plasticity (Ostroff et al., 2002, 2017, Ostroff et al., 2018; Harris et al., 2003; Chirillo et al., 2019; Figure 3C). Similarly, environmental enrichment leads to increased polyribosome association with dendritic spines (Greenough et al., 1985). Consistent with a need for increased protein translation machinery in synaptic development/strengthening, the density of polysome-containing synapses in the developing hippocampus peaks during the first postnatal week of life during a period of active synaptogenesis and synapse maturation (Steward, 1983, 1987; Steward and Falk, 1985, 1986).

While EM analysis reveals changes in polysome number and location during the induction of synaptic plasticity, recent biochemical polysome profiling of isolated hippocampal neuropil suggests that local translation is driven primarily by monosomes, and not polysomes (Biever et al., 2020). Because monosomes are difficult to detect using standard EM staining methods, super-resolution imaging with molecule-specific labeling of ribosomal proteins has been employed to confirm that >75% of inhibitory and excitatory synapses in the mouse hippocampus contain ribosomes (Hafner et al., 2019). These imaging techniques offer promising new approaches to evaluate the extent to which protein translation machinery in nascent synapses is up or down-regulated by changes in neural activity during circuit development (see “Super-Resolution Microscopy” section below).

While important progress has been made investigating links between neural activity, synaptic transmission, and local protein synthesis, key questions remain when considering neural circuit development: to what extent is the specification of the axonal vs. dendritic transcriptome activity-dependent? How do synaptic transmission patterns regulate ribosome engagement with specific mRNA transcripts? Does local, co-translational assembly of protein complexes occur in synapses (Schwarz and Beck, 2019), and if so, does neural activity play a role? New techniques, including site-directed control of protein synthesis (Kim et al., 2020), live imaging of mRNA translation dynamics (Cialek et al., 2020), and advanced ribosome profiling applications (Shiber et al., 2018; Ingolia et al., 2019) will put these and other questions within experimental reach in the coming years.

## Investigating Local Proteomic Networks in Targeted Circuits

While transcript levels in subcellular compartments are often predictive of corresponding protein levels (Zappulo et al., 2017), mRNA-protein relationships can reflect complex spatial and temporal regulation that decouple their respective levels (Liu et al., 2016). Direct comparison of the local transcriptome (RNA-seq) and translatome (Ribo-seq) in hippocampal dendritic neuropil reveals an impressive range (>200-fold difference) in the degree to which an individual mRNA species is engaged with ribosomes (Glock et al., 2020). While transcripts and ribosome footprints covary for most gene products, hundreds of synapse-associated transcripts are locally translated with either increased or decreased efficiency that reflects selective translation initiation (Glock et al., 2020). Transcripts experiencing decreased translational efficiency often show ribosome binding in upstream open reading frames (uORFs) consistent with a role for uORFs in the suppression of protein translation by causing ribosomes to drop off or stall during scanning (Zhang et al., 2019; Glock et al., 2020). In the developing brain, the post-transcriptional regulation of protein production by changes in ribosome engagement plays an essential role in neuronal differentiation by enabling selective activation and repression of translation to guide cell fate determination (Blair et al., 2017; Baser et al., 2019; Rodrigues et al., 2020).

Ribosome stalling is an important regulator of protein synthesis levels (Richter and Coller, 2015; Schuller and Green, 2018) and axon-TRAP experiments have identified ribosome stalling in about 15% of axonal transcripts within developing axons *in situ* (Shigeoka et al., 2016). Specific RNA-binding proteins (RBPs) that cause ribosome stalling may act as translational repressors that confer stability to mRNA/polysome complexes during transport to distal compartments (Graber et al., 2013). After reaching synapses, stalled polyribosomes engaged with mRNA can be reactivated by synaptic transmission to initiate the local translation of synaptic proteins for rapid plasticity of the local proteome (Graber et al., 2013). Important for human brain development and disease, Fragile X mental retardation protein (FMRP) is a critical RBP that drives reversible ribosome stalling during translation of mRNAs essential for normal synaptic development and plasticity (Darnell et al., 2011; Chen et al., 2014; Liu and Cline, 2016; Banerjee et al., 2018).

At the same time, *increased* translational efficiency of certain transcripts may arise from the use of multiple translation initiation sites to enable the production of several unique protein products (Sapkota et al., 2019). The selection of unique initiation sites is further regulated by neural activity, providing yet another opportunity for synaptic transmission to modify local protein production within dynamic connectomes (Sapkota et al., 2019). For both mechanisms (stalling and initiation), it will be of interest to explore whether heterogeneity in the molecular organization of ribosomes confers specificity for the translation of particular mRNAs (Kondrashov et al., 2011; Shi et al., 2017). To investigate the local organization and plasticity of subcompartment proteomes, new methods are being applied to selectively tag and capture nascent proteins and protein-protein interaction networks from genetically-targeted microcircuits.

### Beyond the Translatome: Tools for Investigating Synaptic Proteomes

Similar to assays for isolating the local transcriptome/translatome, biochemical and/or physical dissection techniques enable subcompartment fractions to be isolated for quantitative proteomic analysis. Mass spectrometry analysis performed on synaptic fractions isolated from the tissue has identified major protein components of the synaptic proteome (Laßek et al., 2015; Frank and Grant, 2017; Roy et al., 2018) as well as changes in proteome composition associated with synaptic plasticity (Dieterich and Kreutz, 2016; Baucum, 2017), synapse development (Gonzalez-Lozano et al., 2016), and neurodevelopmental disorders (Kaizuka and Takumi, 2018).

Specificity and resolution have been gained by affinity purification methods, which use antibodies against native proteins, protein biotinylation, or transgenic fusion protein probes (e.g., GFP or affinity tags) to isolate proteins for quantitative analysis (Bauer and Kuster, 2003; Meyer and Selbach, 2015; Dieterich and Kreutz, 2016). These methods, together with post-purification mass spectrometry analyses, have helped identify the proteomic composition of presynaptic vesicles (Chantranupong et al., 2020), cell-surface proteomes (Pischedda et al., 2014; van Oostrum et al., 2020), postsynaptic receptor complexes (Husi et al., 2000; Farr et al., 2004; Fukata et al., 2005; Bartoi et al., 2010; Delint-Ramirez et al., 2010; Von Engelhardt et al., 2010; Schwenk et al., 2012, 2014, 2016; Del Pino et al., 2014), adhesion proteins (Tanaka et al., 2012; Kang et al., 2014; Apóstolo et al., 2020), and scaffolding molecules of excitatory (Dosemeci et al., 2007; Fernández et al., 2009) and inhibitory (Heller et al., 2012) synapses including circuit-specific post-synaptic densities (Selimi et al., 2009). FASS has also been successful in isolating molecularly-defined synaptic proteomes corresponding to specific cell and synapse types (Biesemann et al., 2014; Pfeffer et al., 2020; Figure 2C). Fluorescence sorting and proteomic analysis of developing growth cones have shown that growing axons are enriched with ribosomal proteins and RBPs implicated in the control of local protein translation (Poulopoulos et al., 2019).

**Figure 4:**
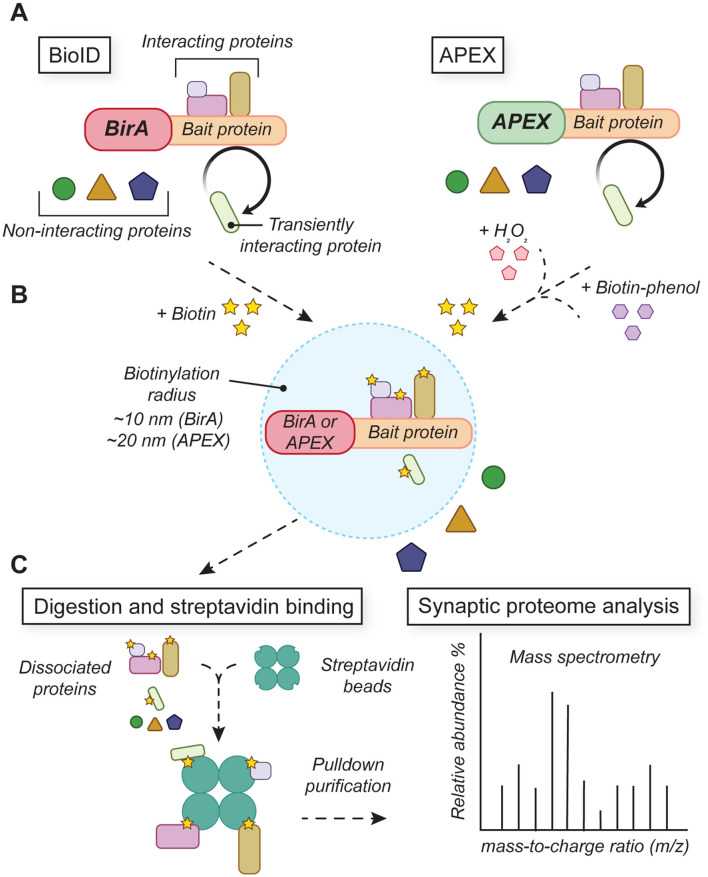
Proximity-labeling tools for analyzing protein networks in developing synapses. **(A)** BioID and APEX tools rely on transgenic fusion constructs that link biotinylation enzymes with target bait proteins. **(B)** Activation of BirA or APEX enzymatic activity drives local biotinylation of proteins that interact with the bait protein. **(C)** Biotinylated proteins are purified and analyzed by mass spectrometry to determine local protein identities and abundances.

To extend the proteomic analysis to subcellular compartments and previously unpurifiable synaptic regions (e.g., the synaptic cleft), genetically-encoded proximity-labeling tools have been developed to selectively tag and purify interacting protein complexes from synapses *in situ* (Han et al., 2018; Wilson and Nairn, 2018; Trinkle-Mulcahy, 2019; Figure 4). Proximity-labeling techniques use enzymatic labeling to biochemically biotinylate local proteins interacting within a short distance (~10–20 nm) of a transgenically expressed peroxidase enzyme (e.g., HRP, APEX, or APEX2; Martell et al., 2012; Lam et al., 2014) or bacterial biotin ligase (e.g., BioID, BioID2, TurboID; Roux et al., 2012; Kim et al., 2016; Branon et al., 2018). By fusing the tagging enzyme to a synaptically-localized protein, other endogenous interacting synaptic proteins are biotinylated and can be immunopurified for subsequent mass spectrometry analysis. Split versions of BioID/TurboID (Cho et al., 2020a, b; Kwak et al., 2020), APEX2 (Han et al., 2019), and HRP (Martell et al., 2016) have been developed to improve labeling specificity, increase the signal-to-noise ratio, and provide greater access to challenging targets.

Proximity-labeling experiments with APEX2 fused to presynaptic terminal protein alpha-synuclein identified hundreds of interactors including synaptic proteins, vesicle trafficking proteins, RBPs, and translation initiation factors important for local protein synthesis (Chung et al., 2017). Similarly, proximity-labeling with HRP targeted to the synaptic cleft revealed uncharacterized “orphan” synaptic proteins in excitatory and inhibitory synapses, including poorly-characterized synapse-enriched proteins essential for synaptogenesis during development (Loh et al., 2016; Cijsouw et al., 2018). Proximity-labeling probes have also been targeted to proteins at the axon initial segment (AIS) to identify previously uncharacterized proteins with important functions in AIS assembly during development (Hamdan et al., 2020).

In addition to the study of protein-protein interactions at synapses and other subcellular compartments, proximity-labeling approaches provide new opportunities to identify protein machinery essential for mRNA processing, trafficking, and translation. The proteome of mRNA-processing organelles (P-bodies and stress granules) has been mapped both with BioID (Youn et al., 2018) and APEX2 (Markmiller et al., 2018; Padrón et al., 2019). When comparing these two approaches, an important advantage of APEX is its ability to directly biotinylate both mRNA and protein. This has led to the development of an APEX-seq strategy for isolating compartment-specific mRNAs together with proteins that are each tagged based on their proximity to the proximity-labeling enzyme (Fazal et al., 2019; Padrón et al., 2019). Using subcellular compartment-specific transgenic proximity-labeling constructs, APEX-seq has revealed a high level of spatial precision in the localization of specific transcripts within unique organelles (Fazal et al., 2019; Padrón et al., 2019). Together with APEX proteomic analysis, this is a promising strategy for future combinatorial transcriptomic/proteomic analysis of mRNAs and mRNA-associated regulatory proteins within developing neurites and synapses.

Building on these successes, proximity-labeling approaches are being extended to the study of synaptic proteomic networks labeled *in vivo*. BioID has successfully captured protein networks at inhibitory and excitatory synapses labeled by viral transfection with postsynaptic excitatory (PSD-95) or inhibitory (gephyrin) scaffolding molecules conjugated to BirA, the promiscuous biotin ligase (Uezu et al., 2016). This *in vivo* labeling strategy found known excitatory/inhibitory synaptic proteins as well as many previously uncharacterized synaptic proteins with essential roles in excitatory synapse development (Uezu et al., 2016). By targeting BirA expression to developing dendritic filopodia and nascent spines, BioID has also revealed 60 candidate proteins associated with dendritic spine development, including previously uncharacterized CARMIL3 involved in actin remodeling essential for spine maturation (Spence et al., 2019). BioID mapping of the presynaptic cytomatrix has been achieved using BirA-Synapsin1a constructs to identify novel cytoskeletal-associated proteins contributing to the regulation of synaptic vesicle dynamics (Dube et al., 2020). To enable the proteomic analysis of specific synaptic connections, split constructs were delivered to glia and neurons to map the extracellular proteome of neuron-astrocyte junctions *in vivo* and identify a novel tripartite adhesion molecule NrCAM essential for inhibitory synaptic development (Takano et al., 2020).

To identify additional regulators of synapse development, cell-surface protein proximity-labeling using horseradish peroxidase (HRP) was used to study the developmental maturation of olfactory projection neurons (OPNs; Li et al., 2020). Mass spectrometry analysis revealed a significant developmental shift in the enrichment of the cell surface proteome from developmental regulators of wiring expressed by young neurons to channels, receptors, and synaptic proteins expressed at maturity (Li et al., 2020). In addition to identifying all previously known cell-surface proteins involved in olfactory circuit wiring, proximity-labeling experiments identified 20 cell-surface proteins not previously implicated in circuit development (Li et al., 2020). These results highlight the power of proximity-labeling technologies for identifying unexpected regulators of cell-cell interactions underlying synaptic development.

As the list of neuronal-compartment-specific proteomic analyses across species and brain regions grows, efforts are underway to curate and expand comprehensive, searchable proteomic databases cataloging the molecular composition and stoichiometry of synaptic connections (Croning et al., 2009; Pielot et al., 2012; von Eichborn et al., 2013). Synapse-specific gene ontology classification using expert-curated annotations has identified >1,100 synaptic genes and defects in many of these genes are associated with neurodevelopmental disorders (Koopmans et al., 2019). A future challenge is to expand such databases to include cell-type and synapse-type-specific information as well as ontology terms that capture key processes in synaptogenesis and synaptic plasticity.

### Investigating Nascent Protein Synthesis in Subcellular Compartments

Because protein turnover helps set the tempo for embryonic development, changes in turnover rate may have important impacts on synaptogenesis and plasticity (Alvarez-Castelao and Schuman, 2015; Matsuda et al., 2020; Rayon et al., 2020). To investigate the nascent synaptic proteome and protein metabolic turnover, newly synthesized proteins can be selectively tagged during the translation process (Cohen and Ziv, 2019). In an approach called Stable Isotope Labeling in Mammals (SILAM), heavy isotopes are incorporated into growing peptide chains *in vivo* (McClatchy et al., 2007a,b). Isotope labeling experiments *in vivo* show that protein turnover is slower in the brain compared with other tissues and many neuronal proteins, including synaptic proteins, are long-lived relative to the average protein half-life (Price et al., 2010; Toyama et al., 2013; Fornasiero et al., 2018; Heo et al., 2018). Important for neural development and activity-dependent plasticity, the metabolic turnover rate for many synaptic proteins is increased by exposure to novel environmental stimuli, consistent with proteome remodeling during synaptic plasticity (Fornasiero et al., 2018; Heo et al., 2018). In the future, it will be of special interest to address whether the turnover rate for select proteins in subcellular compartments is modified during development to promote cell-cell signaling underlying growth and plasticity.

In addition to isotope labeling for mass spectrometry analysis, several alternative labeling approaches have been developed for imaging the spatial location of nascent proteins. For *in situ* analysis of newly translated proteins the antibiotic puromycin can be used as a label that transfers to the growing peptide chain enabling visualization of nascent proteins by direct conjugation with fluorescent dyes (Starck et al., 2004), immunolabeling with anti-puromycin antibodies (Schmidt et al., 2009), or click chemistry-mediated fluorescent labeling using alkyne-functionalized puromycin analogs (Liu et al., 2012). Puromycin labeling (Aviner, 2020) has been used to detect protein synthesis in a variety of subcellular compartments including synapses (Scarnati et al., 2018; Hafner et al., 2019), axons (Batista et al., 2017; Wong et al., 2017), dendrites (Smith et al., 2005; Hafner et al., 2019; Rangaraju et al., 2019), and peripheral astrocytic processes (Sakers et al., 2017). Despite these successful applications, recent evidence suggests that puromycylated peptide chains are released from ribosomes even in the presence of elongation inhibitors, raising the possibility that labeled nascent proteins may diffuse or be trafficked away from the initial site of translation (Enam et al., 2020; Hobson et al., 2020). In this context, additional validation experiments and careful controls are important when interpreting spatial results obtained from puromycylation methods. Future advances in the development of photocleavable caged protein-tagging reagents may increase the spatiotemporal resolution of nascent peptide labeling (Adelmund et al., 2018; Elamri et al., 2018).

As an alternative to puromycin labeling, nascent proteins can be tagged by introducing noncanonical AAs that are incorporated into the growing polypeptide chain (Hinz et al., 2013; Saleh et al., 2019). Using click chemistry labeling (Parker and Pratt, 2020), noncanonical AAs can be biotinylated for pull-down purification/mass spectrometry or fluorescently labeled for microscopic visualization. These approaches, called bio-orthogonal noncanonical amino acid tagging (BONCAT; Dieterich et al., 2006) and fluorescent noncanonical amino acid tagging (FUNCAT; Dieterich et al., 2010) respectively, have been used to measure changes in protein synthesis in response to neurotrophic factors (Dieterich et al., 2010; Genheden et al., 2015; Bowling et al., 2016), dopaminergic transmission (Hodas et al., 2012), activity-dependent homeostatic plasticity (Schanzenbächer et al., 2016, 2018), as well as the induction of long-term synaptic depression (Younts et al., 2016; van Gelder et al., 2020). BONCAT/FUNCAT have also been used to label whole organisms and demonstrate neural activity-dependent changes in protein translation during larval zebrafish (Hinz et al., 2012) and frog development (Shen et al., 2014; Liu and Cline, 2016; Liu et al., 2018).

To extend these approaches *in vivo*, genetic models have been developed for cell-type-specific expression of a mutant methionyl-tRNA synthetase enzyme enabling the selective incorporation of noncanonical AAs in unique neurons and circuits (Hinz et al., 2013; Saleh et al., 2019). Cell-specific noncanonical AA labeling *in vivo* has enabled nascent protein detection in *C. elegans* neurons (Yuet et al., 2015), developing neurons of larval *Drosophila* (Erdmann et al., 2015; Niehues et al., 2015), activity-dependent protein translation in developing zebrafish (Shahar and Schuman, 2020), and experience-dependent changes in excitatory neuron proteomes in the mammalian hippocampus (Alvarez-Castelao et al., 2017) including changes associated with the formation of long-term spatial memory (Evans et al., 2020). While steady progress continues to advance techniques for nascent protein tagging and imaging, challenges remain for developing targeted loss-of-function experiments designed to perturb local protein synthesis. Future technical improvements toward precise spatiotemporal activation of protein synthesis inhibitors will enable direct loss-of-function perturbation of local protein synthesis during synaptogenesis and circuit development (Heumüller et al., 2019).

## Investigating Circuit Organization at the Nanoscale With Super-Resolution Microscopy

To connect transcriptomic and proteomic analyses to structural synaptic development and plasticity, new tools are essential to visualize the spatiotemporal organization of synaptic protein and RNA targets within subcellular compartments. Electron microscopy (EM) provides unparalleled image resolution for synapse analysis but is difficult to combine with conventional molecular labeling tools due to challenges imposed by the use of strong chemical fixatives and epoxy resins in the sample preparation. Further development of EM-compatible transgenic labeling tools will be useful for identifying specific molecular targets and cell/synapse types in EM images (Gustincich et al., 1997; Dubois et al., 2001; Li et al., 2010; Shu et al., 2011; Martell et al., 2012; Atasoy et al., 2014; Paez-Segala et al., 2015; Viswanathan et al., 2015).

Complementary to EM-based approaches, light microscopy enables live imaging and routine investigation of molecular targets using a toolbox of fluorescent proteins, dyes, and synthetic probes. Continued advances in both theory and technology have resulted in the rapid evolution of super-resolution imaging capabilities for molecular analysis of biomolecular targets with nanoscale spatial precision (Sahl et al., 2017; Sigal et al., 2018; Schermelleh et al., 2019). Several types of far-field super-resolution imaging methods have been applied successfully *in situ*: (1) patterned excitation approaches [e.g., STimulated Emission Depletion (STED) microscopy (Hell and Wichmann, 1994; Klar et al., 2000), Structured Illumination Microscopy (SIM; Heintzmann and Cremer, 1999; Gustafsson, 2000), and REversable Saturable OpticaL Fluorescence Transitions (RESOLFT; Hofmann et al., 2005)] drive reversable state changes in fluorescent molecules within sub-diffraction-limit volumes to achieve enhanced resolution of neighboring targets; (2) single-molecule localization microscopy (SMLM) methods (e.g., STochastic Optical Reconstruction Microscopy (STORM; Rust et al., 2006), photoactivated localization microscopy (PALM/fPALM; Betzig et al., 2006; Hess et al., 2006), and Point Accumulation for Imaging in Nanoscale Topography (PAINT; Sharonov and Hochstrasser, 2006) each achieve super-resolution by detecting/localizing the nanoscale position of independent neighboring molecules stochastically over time; and (3) physical Expansion Microscopy (ExM) methods that use swellable polymers to enlarge biological samples and thereby increase the distance between neighboring molecules for enhanced resolution using conventional microscopes (Chen F. et al., 2015).

Applications of super-resolution imaging for studying neuronal and synaptic properties have been reviewed previously (Maglione and Sigrist, 2013; Heller and Rusakov, 2017; Igarashi et al., 2018; Gramlich and Klyachko, 2019; Wassie et al., 2019; Yang and Specht, 2019; Groc and Choquet, 2020; Nosov et al., 2020) and in the following sections, we focus our discussion of super-resolution imaging primarily on key examples applied to the analysis of neurodevelopmental processes *in situ* and *in vivo*. We highlight advanced methods for volumetric super-resolution imaging of developing model organisms, hybrid super-resolution imaging applications for increased spatial resolution, and multiplexed labeling strategies for combined transcriptomic/proteomic analysis in developing neuropil.

### Super-Resolution Imaging of Neural Development and Plasticity in Multiple Model Systems

**Figure 5:**
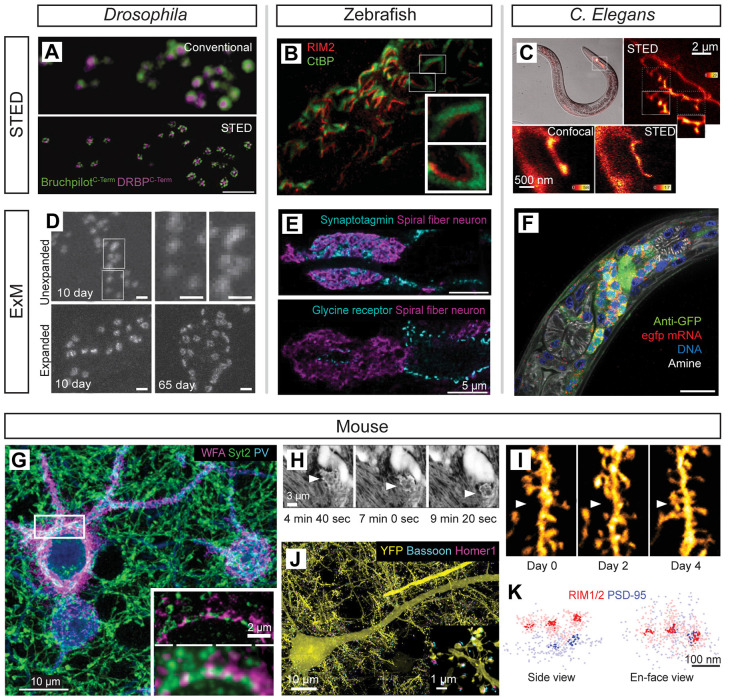
Super-resolution imaging in model systems for studying neural development and plasticity. **(A)** Two-color STED imaging of the neuromuscular junction (NMJ) proteins Bruchpilot (green) and DRBP (magenta) in *Drosophila*; modified from Liu et al. (2011) with permission from AAAS. **(B)** Two-color STED imaging of presynaptic ribbon synapse proteins RIM2 (red) and CtBP (green) in larval Zebrafish photoreceptors; modified from Lv et al. (2012) with permission from Cambridge University Press. **(C)** STED imaging of GFP-labeled neurites in serotonergic neurons of live *C. elegans* highlights the enhanced resolution of STED vs. confocal imaging of neurite branching patterns (insets); modified from Rankin et al. (2011) with permission from Elsevier. **(D)** ExM imaging of Bruchpilot protein reveals developmental refinement of NMJ synapses in 10 days vs. 65-day-old *Drosophila*; modified from Jiang et al. (2018). **(E)** ExM imaging resolves presynaptic Synaptotagmin (blue; top panel) and postsynaptic glycine receptor (blue; bottom panel) organization associated with Kaede-labeled spiral fiber neurons (magenta) in larval Zebrafish hindbrain circuitry; modified from Freifeld et al. (2017). **(F)** Four-color DNA/RNA/protein imaging by ExM in *C. elegans* reveals pan-neuronal GFP in the nerve ring (green) together with GFP mRNA labeling (red), DAPI stain (blue), and Atto647N conjugated NHS-ester for amine labeling (white); modified from Yu et al. (2020). **(G)** Volumetric STORM imaging of developing perineuronal nets and synaptic connections revealed by labeling of wisteria floribunda agglutinin (magenta), Synaptotagmin-2 (green), and parvalbumin (cyan) proteins in the mouse cortex; insets highlight the resolution improvement of STORM (top inset) compared to diffraction-limited imaging (bottom inset); modified from Sigal et al. (2019). **(H)** Super-resolution shadow imaging (SUSHI) reveals microglial dynamics (arrowheads) in living mouse brain slices; modified from Tønnesen et al. (2018) with permission from Elsevier. **(I)** 2P-STED imaging of YFP-labeled neurites reveals spine dynamics over multiple days *in vivo* (arrowheads) in the mouse hippocampus; modified from Pfeiffer et al. (2018). **(J)** Lattice light-sheet imaging of expanded mouse cortical tissue reveals synaptic connections over large volumes. Antibody labeling for presynaptic bassoon (cyan), postsynaptic Homer1 (magenta), and neurons (YFP) is shown; modified from Gao et al. (2019a) with permission from AAAS. **(K)** Subsynaptic nanocolumns of aligned presynaptic (RIM1/2, red) and postsynaptic (PSD95, blue) proteins imaged in mouse brain tissue by STORM; modified from Tang et al. (2016) with permission from Springer Nature.

Super-resolution microscopy techniques have been important tools for the study of circuit development and structural plasticity in several model organisms including *Drosophila* (Figures 5A,D), zebrafish (Figures 5B,E), *C. elegans* (Figures 5C,F), and mouse (Figures 5G–K). An early application *in situ* was the use of STED imaging to reveal a ring-like subsynaptic organization of Bruchpilot (BRP), a structural protein at the *Drosophila* neuromuscular junction (NMJ) essential for the developmental positioning of Ca^2+^ channels and synaptic vesicles (Kittel et al., 2006). Subsequent super-resolution investigations of this synapse demonstrated an essential role for Rab3-interacting molecule (RIM)-binding proteins in the formation/function of the NMJ while providing additional quantitative nanoscale measurements of the relationships between presynaptic Ca^2+^ channels and essential active zone proteins (Liu et al., 2011; Figure 5A). Developmental studies of the NMJ using ExM imaging show a progressive increase in BRP area and complexity as the synapse matures (Jiang et al., 2018; Figure 5D). STED and STORM have further characterized the rapid remodeling of BRP and other active zone components during synaptic plasticity, showing that the presynaptic proteins regulating transmission are modulated to maintain synaptic efficacy during homeostatic scaling (Weyhersmüller et al., 2011; Böhme et al., 2019; Mrestani et al., 2019). STORM imaging of the NMJ has further resolved individual BRP clusters within rings at the active zone (Ehmann et al., 2014) and was later used to discover species differences in subsynaptic NMJ active zone organization in mouse vs. humans (Jones et al., 2017). Additional nanoscale imaging experiments demonstrated essential relationships amongst active-zone organizing proteins including Liprin-alpha, Syd-1, neurexin, and UNC13 SNARE proteins (Fouquet et al., 2009; Owald et al., 2010, 2012; Jepson et al., 2014; Muhammad et al., 2015; Böhme et al., 2016; Goel et al., 2019; Petzoldt et al., 2020) underlying synaptogenesis and synaptic structural/functional organization at the NMJ (Ghelani and Sigrist, 2018).

For examining central synapses, several super-resolution approaches have been applied to imaging neural tissue from mature and developing brains. The first example of synapse imaging *in situ* used STochastic Optical Reconstruction Microscopy (STORM) to reconstruct the relative spatial positions of pre/postsynaptic proteins in thin cryosections of brain tissue to map synaptic structure with molecular specificity and nanoscale resolution (Dani et al., 2010). Similar SMLM imaging in brain tissue has been performed *in situ* to characterize synaptic/neuronal structure (Beaudoin et al., 2012; Specht et al., 2013; Xu et al., 2013; Viswanathan et al., 2015; Chamma et al., 2016; Spühler et al., 2016; Bednarz et al., 2020) including defects in synaptogenesis resulting from perturbations of adhesion molecule complex formation (Beaudoin et al., 2012), changes in synaptic protein organization during hormonal release cycles (Bednarz et al., 2020), and synapse-specific differences in subsynaptic domains (Broadhead et al., 2016).

STORM imaging enabled the discovery of aligned nanocolumns of clustered presynaptic release machinery and postsynaptic receptors apposed across the synaptic cleft, an arrangement that promotes efficient neurotransmission (Figure 5K; Tang et al., 2016). These subsynaptic domains (SSDs) have been further investigated using STED (Nair et al., 2013; Broadhead et al., 2016; Dzyubenko et al., 2016; Hruska et al., 2018; Masch et al., 2018; Wegner et al., 2018; Wiesner et al., 2020), SMLM (MacGillavry et al., 2013; Nair et al., 2013; Specht et al., 2013; Broadhead et al., 2016; Pennacchietti et al., 2017; Sinnen et al., 2017; Kellermayer et al., 2018; Inavalli et al., 2019; Ferreira et al., 2020; Goncalves et al., 2020; Yang et al., 2020), PAINT (Nair et al., 2013), and SIM (Crosby et al., 2019). Subsynaptic clusters of glutamatergic receptors are developmentally-regulated (Kellermayer et al., 2018) and SSDs containing receptors and critical synaptic machinery show activity-dependent changes in response to stimulation protocols for inducing synaptic potentiation, depression, and homeostatic plasticity (MacGillavry et al., 2013; Tang et al., 2016; Pennacchietti et al., 2017; Sinnen et al., 2017; Ferreira et al., 2020; Wiesner et al., 2020; Yang et al., 2020). Because SSD and nanocolumn remodeling may be a critical driver of synaptic plasticity (Chen et al., 2018; Yang and Specht, 2019), it will be of considerable interest to explore the development and activity-dependent refinement of synaptic proteins in SSDs in specific microcircuits during synaptogenesis and synapse maturation.

Super-resolution imaging has further enabled the analysis of neurite/cytoskeletal organization and plasticity in developing circuits. STORM imaging *in situ* was used to discover a periodic cytoskeletal structure in neurites (Xu et al., 2013) which has been identified in other brain regions using SIM (He et al., 2016; Schlüter et al., 2019), and STED (Schlüter et al., 2019). A recent study used STED imaging to show the developmental stability of this membrane periodic skeleton in the AIS of developing cochlear nucleus neurons (Akter et al., 2020). STED imaging has helped characterize additional cytoskeletal organization including the developmental refinement of a ring-like pattern of the cytoskeletal protein spectrin during auditory hair cell maturation (Liu et al., 2019). For examining cytoskeletal changes in postsynaptic neurites, STED imaging has proven particularly effective for the analysis of spine development *in situ*. STED microscopy revealed that spine heads/necks become smaller/wider respectively over development, indicating reduced synaptic compartmentalization during synaptic maturation (Wijetunge et al., 2014).

Spine imaging with SIM (Turcotte et al., 2019) as well as with STED/RESOLFT has been extended to imaging live spine dynamics *in situ*/*in vivo* (Nägerl et al., 2008; Grotjohann et al., 2011; Berning et al., 2012; Testa et al., 2012; Bethge et al., 2013; Willig et al., 2014; Schneider et al., 2015; Wegner et al., 2017, 2020; Arizono et al., 2020; Steffens et al., 2020). *In vivo* super-resolution imaging highlights diverse postsynaptic density (PSD95) organization (Masch et al., 2018) and dynamics (Wegner et al., 2018), including the high turnover of small hippocampal spines over multiple days of imaging (Pfeiffer et al., 2018; Figure 5I). LTP induction by local synaptic stimulation causes a coordinated increase/decrease in spine head size and neck length that each contributes to a change in synaptic weighting (Tønnesen et al., 2014). Live STED and fixed-tissue STORM provided imaging evidence showing that LTP induction also causes a withdrawal of peripheral astrocytic processes from synapses as part of a mechanism that increases glutamate spillover to influence neighboring spines (Henneberger et al., 2020).

In a clever advance, labeling the extracellular space in brain tissue with a membrane-impermeant diffusible fluorophore has enabled live STED imaging of synaptic neuropil in a technique called super-resolution shadow imaging (SUSHI; Tønnesen et al., 2018). SUSHI revealed rapid, activity-dependent remodeling of the extracellular space around dendritic spines following synapse activation as well as dynamic microglial motility within the neuropil (Figure 5H). Considering essential glial contributions to synaptic refinement in developing circuits (Paolicelli et al., 2011; Wilton et al., 2019; Cheadle et al., 2020), SUSHI is a promising approach for quantifying dynamic glia-synapse interactions underlying synaptogenesis and synaptic pruning in living brain tissue. In a separate application of live STED imaging, time-lapse recordings showed dynamic activity-dependent swelling of axon shafts and synaptic boutons that influenced action potential conduction delay (Chéreau et al., 2017). Such tools offer an opportunity to explore whether changes in activity-dependent axon shaft caliber caused by spontaneous and sensory-evoked activity impact spike-timing-dependent plasticity during neural development. Super-resolution imaging has also been combined with live electrophysiological recording techniques and intracellular labeling, enabling new experiments designed to investigate nanoscale remodeling of targeted synapses following *in situ* manipulations of molecular signaling or neural activity (Dudok et al., 2015; Barna et al., 2016).

In contrast to other super-resolution techniques, ExM is not compatible with live imaging but has the advantage of enabling volumetric nanoscale imaging using conventional fluorescence microscopes. The first demonstration of ExM used physical expansion and immunohistochemical labeling to demonstrate synaptic imaging over 3D volumes of mouse hippocampal tissue with an ~4× improvement in image resolution over conventional confocal imaging (Chen F. et al., 2015). ExM resolution can be further increased using an iterative expansion approach with serial expansion steps (Chang et al., 2017). The development of more homogeneous hydrogels may improve ExM image resolution by minimizing physical distortions caused by irregularities in hydrogel polymerization (Gao et al., 2019a,b). To further boost ExM throughput, expanded samples have been imaged by lattice-light-sheet microscopy enabling the reconstruction of transgenically-labeled neurons and synaptic connections across a cortical column in the mouse and entire *Drosophila* brains with sub-diffraction-limit resolution (Gao et al., 2019a; Figure 5J). For ease-of-use and increased application in diverse biological contexts, ExM labeling methods have been simplified (Chozinski et al., 2016; Tillberg et al., 2016) and optimized protocols have been established for applications in model systems important for developmental analysis including zebrafish (Freifeld et al., 2017), *C. elegans* (Yu et al., 2020), and *Drosophila* (Jiang et al., 2018; Gao et al., 2019a; Figures 5D–F). ExM has revealed subtle defects in the developing fly (Menon et al., 2019) and mouse visual system (Burger et al., 2020) in genetic loss-of-function deletion experiments impacting synaptogenesis.

### Volumetric Super-Resolution Imaging and Hybrid Methods for Increased Spatial Resolution

SMLM experiments have often been limited to 1–2 μm of axial depth due to challenges associated with reduced signal-to-noise, fluorophore bleaching, and aberrations when imaging deeper in tissue. Advances in SMLM using adaptive optics (Mlodzianoski et al., 2018) and 3D point-spread function modeling (Xu et al., 2020) have enabled SMLM tools to image deeper in tissue, but the limited photon budget of fluorescent proteins and organic dyes together with optical scattering/aberrations present challenges for routine large-volume tissue imaging. To address these challenges, volumetric super-resolution imaging has been achieved *via* ultrathin sample sectioning for tomographic three-dimensional image reconstruction using SMLM (Nanguneri et al., 2012; Sigal et al., 2015) and STED techniques (Punge et al., 2008; Holderith et al., 2020). Serial-section approaches overcome many of the difficulties associated with scattering, bleaching, background, optical aberrations, and buffer penetration when imaging thick specimens (Sigal et al., 2015). This methodology was used to investigate activity-dependent maturation and plasticity of perineuronal nets and synaptic connections associated with parvalbumin-positive (PV+) interneurons in the mouse cortex (Sigal et al., 2019; Figure 5G).

For larger volume reconstructions, ExM has been combined with lattice-light-sheet microscopy for high-throughput imaging of neural circuits including entire fly brains (Gao et al., 2019a; Figure 5J). To further increase image resolution, Ex-SIM (Cahoon et al., 2017; Halpern et al., 2017), Ex-STED (Gao et al., 2018), and Ex-STORM (Cang et al., 2016; Shi et al., 2019) have each combined physical expansion with optical super-resolution imaging to achieve multiplicative increases in spatial resolution. By combining the advantages of single-molecule photoswitching and spatially-patterned excitation, MINFLUX achieves the highest resolution reported in far-field fluorescence imaging by using a central excitation minimum to localize individual molecules based on their minimal fluorescence emission (Balzarotti et al., 2017). MINFLUX has been used to identify postsynaptic protein clusters with an image resolution at the true molecular-scale (2–3 nm; Gwosch et al., 2020). Related hybrid SIM + SMLM implementations that use diffraction-limited standing wave patterns have enabled molecule detection with doubled localization precision (Gu et al., 2019; Cnossen et al., 2020), thereby demonstrating opportunities for extending hybrid approaches to larger fields of view. As super-resolution imaging hardware and theory advance, achieving molecular-scale spatial resolution will require innovative solutions to increase sample labeling efficiency and enable target-specific detection without the use of bulky probes (e.g., antibodies) that create size artifacts in nanoscale imaging (Li and Vaughan, 2018; Gwosch et al., 2020).

### Combining Nanoscale Spatial Transcriptomic and Proteomic Imaging in Circuits

**Figure 6:**
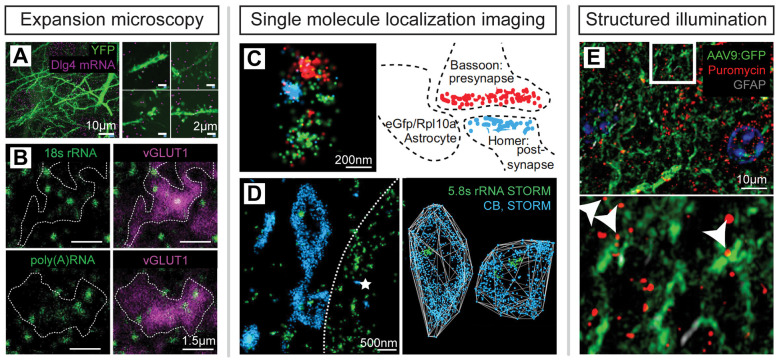
Super-resolution imaging of RNA, ribosomes and nascent proteins *in situ*. **(A)** ExM reveals mRNA in synaptic neuropil; modified from Chen et al. (2016) with permission from Springer Nature. **(B)** ExM imaging of ribosomal RNA and mRNA in excitatory synapses in the mouse brain; modified from Hafner et al. (2019) with permission from AAAS. **(C)** STORM imaging of GFP-tagged ribosomes in peripheral astrocytic processes adjacent to synapses; modified from Sakers et al. (2017). **(D)** STORM imaging identifies ribosomal RNA within interneuron axon terminals defined by endocannabinoid receptor (CB1) expression in mouse hippocampal tissue. The dotted line delineates the neighboring, postsynaptic cell soma (star); modified from Younts et al. (2016) with permission from Elsevier. **(E)** Puromycin labeling reveals nascent protein synthesis in peripheral astrocytic processes imaged by SIM; modified from Sakers et al. (2017).

RNA-seq and mass spectrometry analyses provide rich databases identifying thousands of species of molecules in varying abundances in synaptic neuropil but are limited in their ability to provide a spatial context for specific molecular interactions. Super-resolution microscopy is complementary in that it provides spatial location information about RNA and proteins and can also generate corroborating measurements of target abundance using molecular counting techniques (Sugiyama et al., 2005; Specht et al., 2013; Jungmann et al., 2016; Cella Zanacchi et al., 2019). In support of biochemical evidence for local protein translation in subcellular compartments, super-resolution imaging experiments have confirmed the presence of stalled polysomes in neurites (Graber et al., 2017), ribosomes, and mRNAs in a majority of synapses (Younts et al., 2016; Sakers et al., 2017; Hafner et al., 2019) as well as axons, dendrites, and glial processes adjacent to synapses (Chen et al., 2016; Ouwenga et al., 2017; Sakers et al., 2017), and nascent protein production in dendrites and peripheral glial processes (Sakers et al., 2017; Sun et al., 2020; Figure 6). A central challenge is the future integration of spatial transcriptomic and proteomic imaging for high-throughput spatial mapping of mRNAs and proteins together with nanoscale resolution.

Several multiplexed labeling and target-detection strategies have been developed to address the problem. Array tomography uses multi-round immunohistochemistry (IHC) and antibody elution on ultrathin sectioned brain material to achieve multiplexed proteomic imaging with improved axial image resolution while further enabling correlative electron microscopy (Micheva and Smith, 2007; Micheva et al., 2010). Post-embedding labeling efficiency over multiple rounds of staining can be increased by simple chemical etching of the resin substrate, enabling multiplexed STED imaging to visualize synaptic connections *in situ* (Holderith et al., 2020). Multi-round IHC has also been used with SMLM methods (Tam et al., 2014; Yi et al., 2016) and has been automated to increase experimental throughput (Klevanski et al., 2020). Multi-round IHC is also compatible with ExM to achieve multiplexed synaptic and neuronal imaging (Ku et al., 2016; Shen et al., 2020).

**Figure 7:**
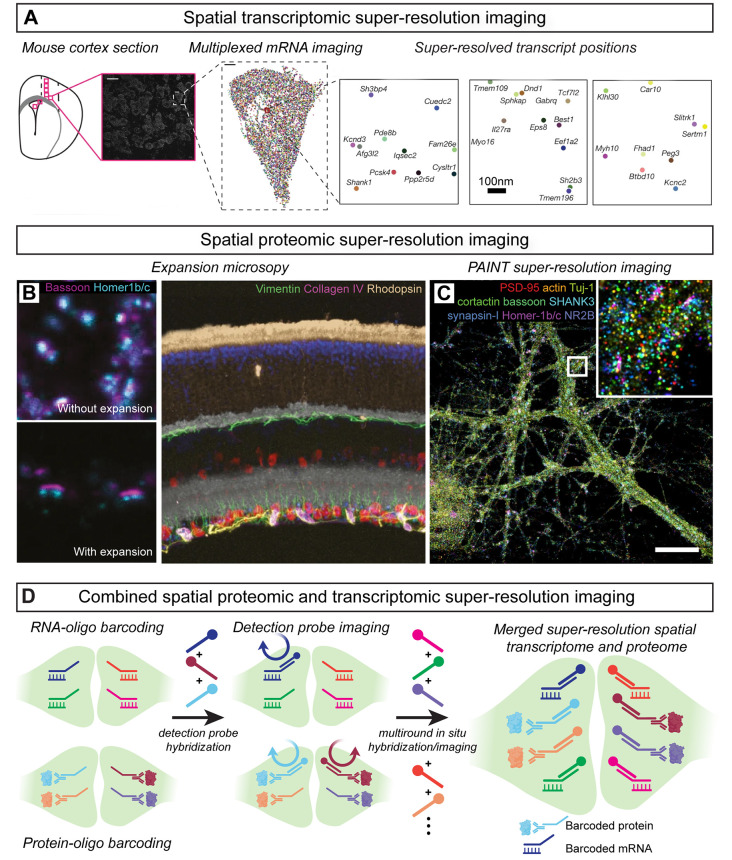
Multiplexed spatial transcriptomic and proteomic imaging *in situ*. **(A)** Spatialtranscriptomic imaging with single-molecule localization identifies mRNA species and position withnanoscale image resolution; modified from Eng et al. (2019) with permission from Springer Nature.**(B)** Immunostaining with signal amplification by exchange reaction (Immuno-SABER) demonstratesmultiplexed proteomic super-resolution imaging in expanded mouse retina; modified from Saka et al. (2019) with permission from Springer Nature. **(C)** Probe-based Imaging for SequentialMultiplexing (PRISM) demonstrates multiplexed SMLM of synaptic proteins *in vitro*; modified fromGuo et al. (2019). **(D)** Nanoscale spatial transcriptomic and proteomic imaging can be combined bybarcoding mRNAs and proteins with unique DNA-oligo tags to determine their position and identityacross multiple rounds of detection probe hybridization and imaging.

Multi-round labeling has also been used to develop spatial transcriptomic approaches for mapping RNA identity and position *in situ* (Lein et al., 2017; Asp et al., 2020). Fluorescence *in situ* RNA sequencing (FISSEQ; Lee et al., 2014) and spatially-resolved transcript amplicon readout mapping (STARmap; Wang X. et al., 2018) each converts cellular mRNAs to barcoded DNA amplicons that are sequenced-by-ligation to determine mRNA identities. STARmap experiments demonstrated the detection of >1,000 transcripts that define the molecular identity and spatial position of unique neuronal populations in the visual cortex and this technique was further used to spatially map activity-dependent changes in gene expression in response to sensory experience (Wang X. et al., 2018). In a separate technical approach, multi-round *in situ* hybridization methods such as multiplexed error-robust fluorescence *in situ* hybridization (MERFISH; Chen K. H. et al., 2015) and sequential fluorescence *in situ* hybridization (seqFISH; Lubeck et al., 2014) use transcript-specific barcode designs to map the identities and spatial positions of RNAs across multiple rounds of detection oligo hybridization. MERFISH achieves volumetric mapping of cell identities and positions defined by unique transcriptional signatures *in situ* and has been further used to identify cell populations that are specifically active during social behaviors (Moffitt et al., 2018; Zhang et al., 2020). Similarly, seqFISH has been used to map hippocampal cell types defined by unique mRNA expression patterns (Shah et al., 2016). To extend multi-round mRNA imaging methods to the nanoscale, multiplexed *in situ* hybridization has been performed with expansion microscopy (Chen et al., 2016; Wang G. et al., 2018) as well as SMLM (Eng et al., 2019) in brain tissue (Figure 7A). Such approaches enable sub-diffraction-limit spatial localization of identified transcripts and pave the way for future spatial transcriptomic mapping of mRNAs pools in subcellular compartments.

In a step toward this goal, multiplexed super-resolution synaptic proteome imaging has been achieved using DNA-oligo-linked primary antibodies which are imaged across multiple rounds of hybridization using complementary detection oligos (Wang et al., 2017; Guo et al., 2019; Saka et al., 2019; Figures 7B,C). Immunostaining with signal amplification by exchange reaction (Immuno-SABER) used DNA-barcoded antibodies for multiplexed protein super-resolution imaging in expanded mouse retinal tissue (Saka et al., 2019; Figure 7B). A related approach, Probe-based Imaging for Sequential Multiplexing (PRISM), used DNA-barcoded primary antibody labeling and sequential oligo probe hybridization to map synaptic proteomes *in vitro* using PAINT (Guo et al., 2019; Figure 7C). In each of these methods, specific protein targets are tagged with unique oligo barcodes making multiplexed proteomic imaging compatible with *in situ* hybridization strategies for spatial transcriptomic imaging (Chen K. H. et al., 2015; Eng et al., 2019; Kishi et al., 2019; Figure 7D). In this way, DNA-based barcode labeling techniques are poised to enable combined multiplexed super-resolution imaging of proteomes and transcriptomes in subcellular compartments of developing circuits. In the future, these approaches may even be combined with DNA microscopy (Weinstein et al., 2019) in expanded hydrogels to achieve optics-free super-resolution spatial transcriptomic/proteomic analysis.

## Discussion

The developmental connectomics strategy presented here enables new experiments to determine the molecular blueprint for synaptic development and plasticity in neural circuits. The application of complementary techniques in transcriptomics, translatomics, proteomics, and super-resolution structural imaging establishes an integrative framework for investigating mechanistic links between mRNA trafficking, local protein synthesis regulation, and changes in subsynaptic molecular organization underlying circuit development.

Molecular sequencing experiments, both at the RNA and protein levels, generate foundational databases identifying regulators of connectome development and plasticity. Also, proteomic (Sharma et al., 2015; Koopmans et al., 2019; Perez-Riverol et al., 2019) and transcriptomic (Ecker et al., 2017; Keil et al., 2018; Solanelles-Farré and Telley, 2021) databases inform hypotheses for gain/loss-of-function studies and probe design/selection for spatial analyses. In a complementary way, spatial transcriptomic databases are resources for validating and mapping spatial gene expression patterns in circuits (Fan et al., 2020). As technical advances increase experimental throughput, a central analytical challenge is the computational integration of multiomic and spatial information within user-friendly environments to extract biological results from big data (Ritchie et al., 2015; Conesa and Beck, 2019; Leonavicius et al., 2019; Leonelli, 2019; Brademan et al., 2020).

Several analytical approaches for integrating multiomic data have been applied to better define cell types in the mature and developing brain (Stuart et al., 2019; Welch et al., 2019; Solanelles-Farré and Telley, 2021). Computational methods such as integrative non-negative matrix factorization (Welch et al., 2019) and canonical correlation analysis (Stuart et al., 2019) are complementary strategies for identifying shared cell types across experiments and data formats including the association of single-cell RNA-seq (scRNA-seq) profiles with spatial transcriptomic maps. These approaches thereby leverage scRNA-seq coverage to predict transcriptome-level spatial expression patterns (Stuart et al., 2019; Welch et al., 2019). As a future development, deep learning shows exciting promise for linking multiomic and spatial data across biological scales and formats (Bersanelli et al., 2016; Haas et al., 2017; Mirza et al., 2019; Nguyen and Wang, 2020). By registering genome-wide single-cell sequencing data to sparse spatial transcriptomic reference frames, deep learning computer vision methods predict spatial expression patterns with increased coverage, error reduction, and multiomic integration (Biancalani et al., 2020; Ma et al., 2020). In principle, similar computational strategies could integrate target-specific proteomic datasets with mass spectrometry imaging (MSI; Xu and Li, 2019) or multi-round protein imaging for spatial proteomics.

Though deep learning methods have thus far been developed for analyzing cell types and spatial organization, related approaches should facilitate integrative analysis of subcompartment-specific multiomic and super-resolution imaging data to characterize molecular networks driving synaptic/neurite maturation and plasticity (Poulopoulos et al., 2019; Chauhan et al., 2020; Glock et al., 2020). To push image-based molecular network analysis to the subcellular scale, deep learning has the potential to automate image optimization and enhance spatial resolution across imaging modalities (LeCun et al., 2015; Belthangady and Royer, 2019). As techniques for multiplexed super-resolution microscopy mature, super-resolution image repositories will serve as training resources for supervised (Kulikov et al., 2019) and unsupervised deep learning algorithms for synapse classification (Wiesner et al., 2020). As elements of an integrative experimental framework, continued advances in multiomic analysis, super-resolution spatial imaging, and deep learning-based data integration are advancing our understanding of the mechanisms driving connectome assembly, activity-dependent refinement, and degeneration in aging and disease.

## Author Contributions

JM and CS wrote the manuscript and approved the submitted version.

## Conflict of Interest

The authors declare that the research was conducted in the absence of any commercial or financial relationships that could be construed as a potential conflict of interest.
